# Supplementary material for: Dual-Responsive Photonic Crystal Sensors Based on Physical Crossing-Linking SF-PNIPAM Dual-Crosslinked Hydrogel
Source: Gels. 2022 May 30;8(6):339. doi: 10.3390/gels8060339 (PMC9223110; doi:10.3390/gels8060339)
Supplement: Supplementary file 1 [file gels-08-00339-s001.zip › Supporting information.pdf]

Supporting information for

Dual-Responsive Photonic Crystal Sensors Based on Physical Crossing-Linking SF-PNIPAM-IPN Hydrogel

Wenxiang Zheng[1], Xiaolu Cai[1], Dan Yan[2,*], Ghulam Murtaza[1], Zihui Meng[1], and Lili Qiu[1,*]

Table S1. The formula of the SF-PNIPAM IPN nanocomposite film

Number	NIPAM (g)	SF (g)	LMSH (g)	APS (g)	TEMED (μL)	H_2O (mL)
1	0.95	0.05	0.18	0.0090	90	10
2	0.90	0.10	0.18	0.0090	90	10
3	0.85	0.15	0.18	0.0090	90	10
4	0.80	0.20	0.18	0.0090	90	10
5	0.85	0.25	0.20	0.0090	90	10
6	1	0.20	0.20	0.0090	90	10
7	1	0.20	0.20	0.0100	100	10
8	1	0.20	0.20	0.0100	100	9

Table S2. Optical properties of the SF-PNIPAM IPN nanocomposite with different pH

Number	pH	Structural Color	Reflection Peak/nm
1	5	Blue	677
2	6	Blue	678
3	7	Blue	677
4	8	Blue	677
5	9	Blue	678
